# Supplementary material for: Myeloid cells interact with a subset of thyrocytes to promote their migration and follicle formation through NF-κB
Source: Nat Commun. 2023 Dec 6;14:8082. doi: 10.1038/s41467-023-43895-8 (PMC10700497; doi:10.1038/s41467-023-43895-8)
Supplement: Supplementary file 1 — Supplementary Information [file 41467_2023_43895_MOESM1_ESM.pdf]

Supplementary Figures and legends

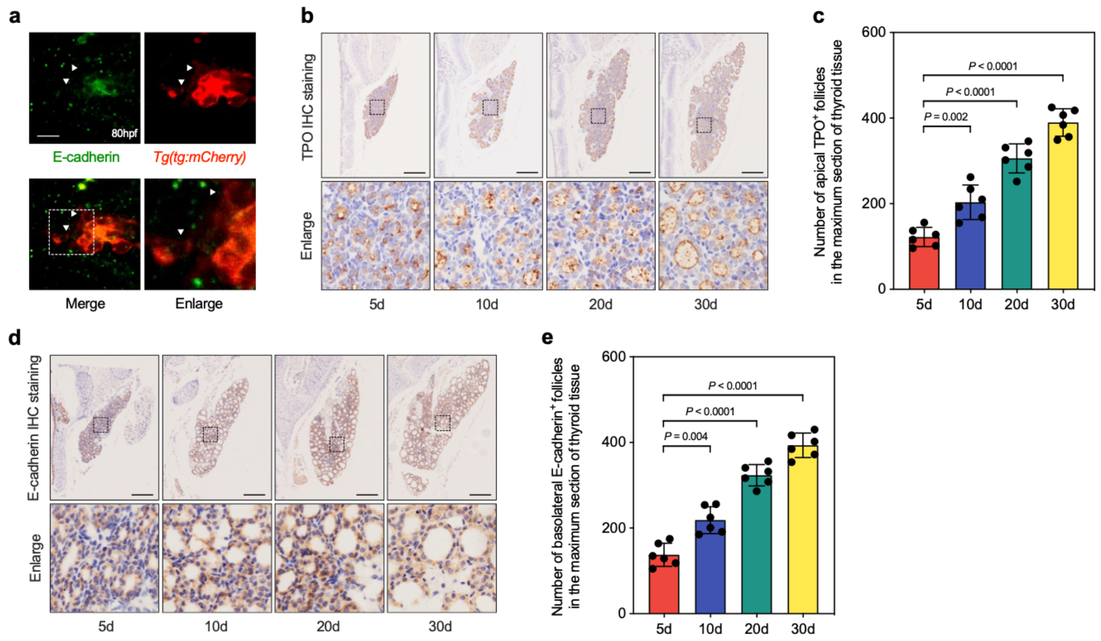

Fig. S1. Heterogenous thyrocytes existed in thyroid tissues of postnatal mice. a, Immunofluorescence assay showing reduced E-cadherin in front thyrocytes extended out of the primordium (marked by arrowheads and framed). **b-c,** Representative images **(b)** showing TPO expression in postnatal day 5, 10, 20, and 30 mice thyroid tissues immunohistochemistry (IHC) analysis. Statistical assessment **(c)** of the number of apical TPO positive lumens with growth. The maximum sections of mice thyroid tissues were included for each group and measured. **d-e**, Representative images **(d)** showing E-cadherin expression in postnatal day 5, 10, 20, and 30 mice thyroid tissues by IHC analysis. Statistical assessment **(e)** of the number of follicles with basolateral membrane E-cadherin expression at varying developmental time examined. Scale bar, 50 μm in **a**, and 100 μm in **b** and **d**. Data are shown as mean ± SD, n = 6 biologically independent samples, statistical significance was determined by One-way ANOVA, followed by Tukey's multiple comparison test (**c**, **e**). Source data are provided as a Source data file. TPO, thyroid peroxidase.

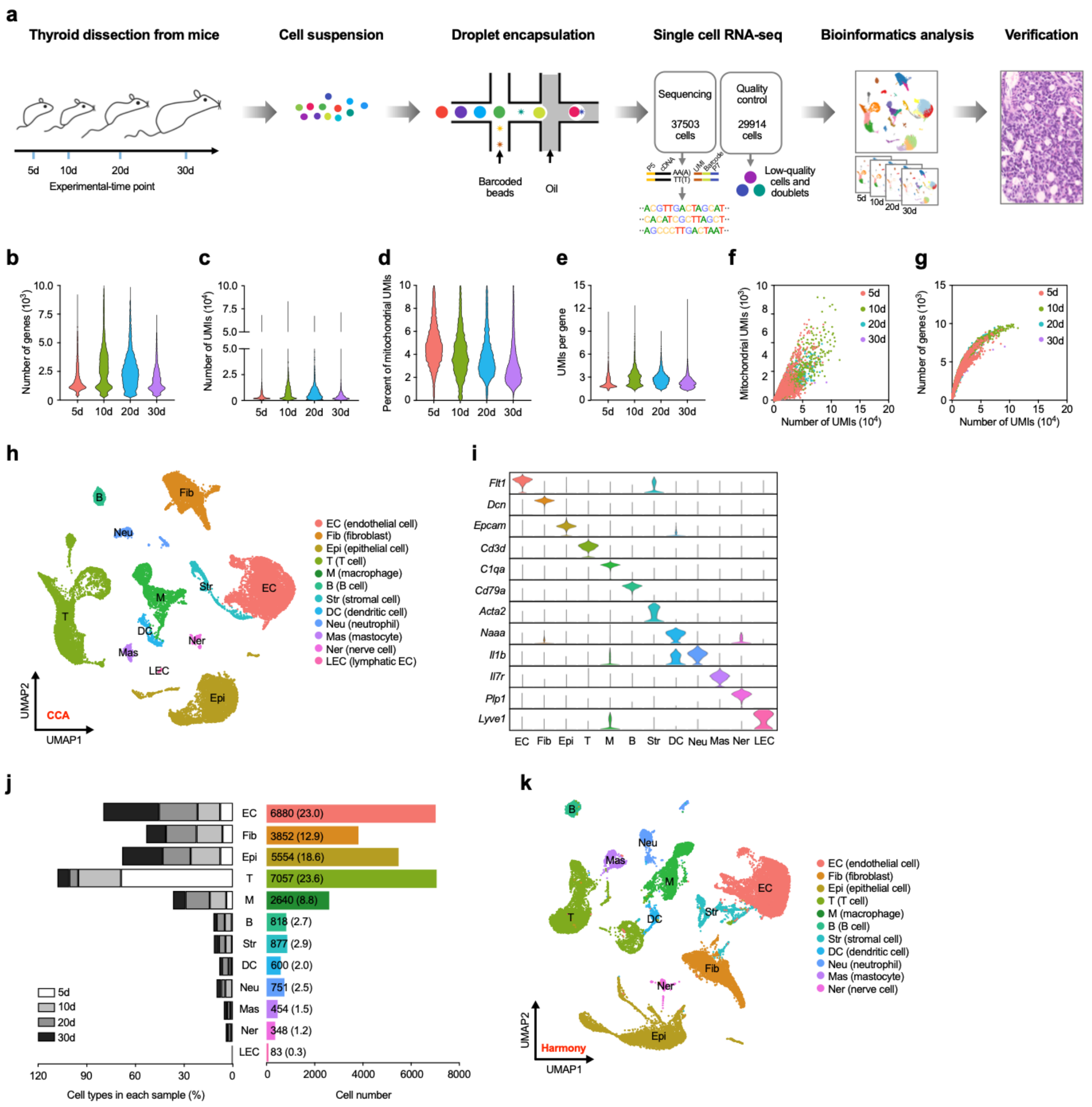

Fig. S2. Major cell types identified from thyroid tissues at postnatal day 5, 10, 20, and 30 mice by 10 × scRNA-seq. a, Workflow showing the 10 × single cell sequencing strategy. Mice thyroid tissues at postnatal day 5, 10, 20 and 30 were collected respectively for scRNA-seq. **b-e,** Sequencing, and quality control metrics for each sample. The distributions maps of each quality control metrics for cells that were retained following removal of low-quality cells. Number of unique genes detected per cell **(b)**, Number of UMIs detected per cell **(c)**, Percentage of UMIs that maps to a mitochondrial protein-encoding gene per cell **(d)**, and UMIs per gene **(e)** detected in each sample were shown. **f-g,** Dot plot showing the relationship between the number of UMIs and mitochondrial UMIs **(f)** or number of genes **(g)** in each sample after quality control. **h,** UMAP plot of all cells collected from the mice thyroid tissues at postnatal day 5, 10, 20 and 30. **i,** Violin plot of expression of canonical marker genes or different

expression genes (DEGs) among identified cell types. **j,** Cell counts of each cell type and percentage among total cells of thyroid tissues at postnatal day 5, 10, 20 and 30 mice (displayed in brackets). **k,** UMAP plot of cell clusters with batch effect correction by Harmony method. Source data are provided as a Source data file. UMI, unique molecular identifier; EC, endothelial cell; Fib, fibroblast; Epi, epithelial cell; T, T lymphocyte; M, macrophage; B, B lymphocyte; Str, stromal cell; DC, dendric cell; Mas, mastocyte; Ner, nervous cell; LEC, lymphatic endothelial cell; scRNA-seq, single cell RNA sequencing.

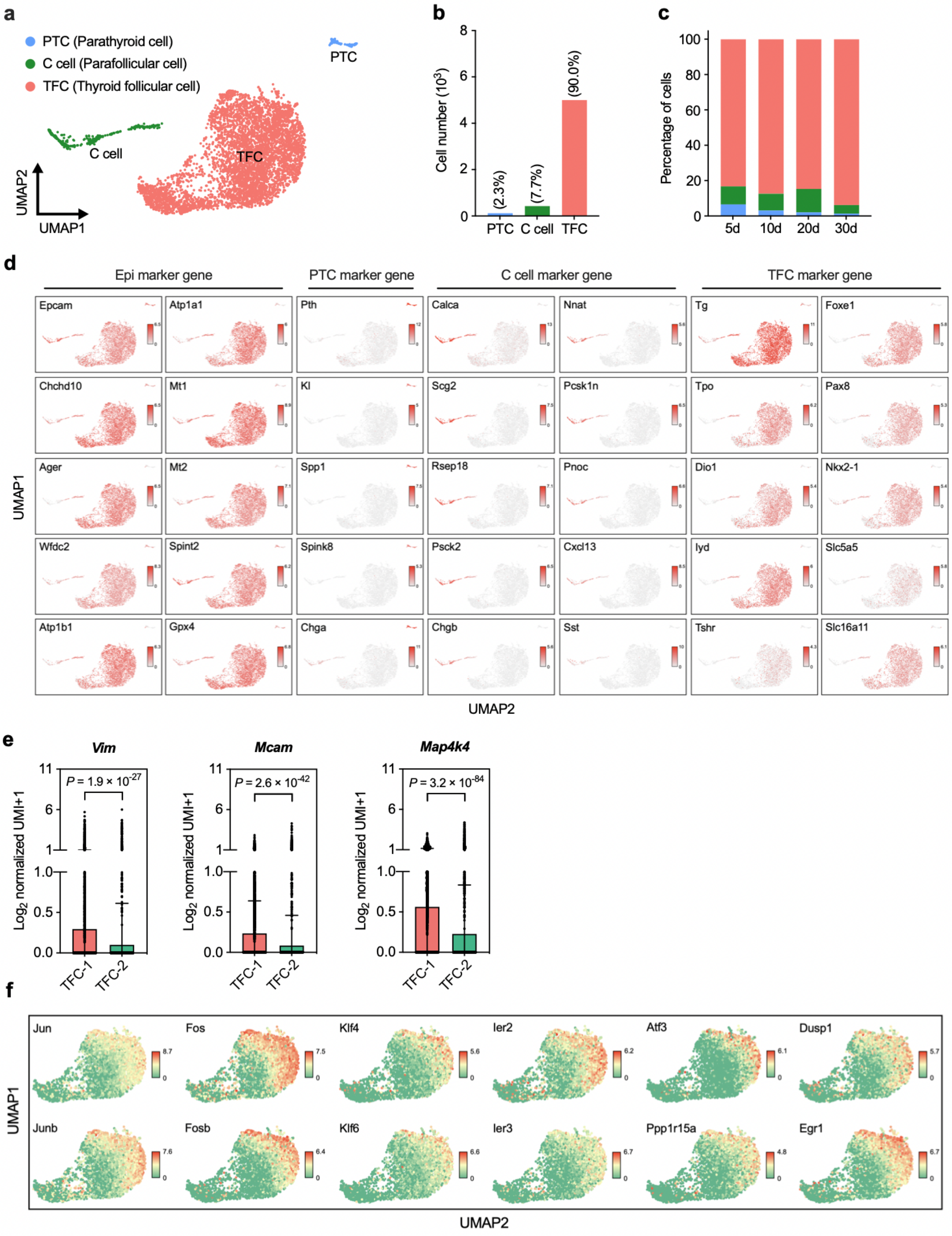

Fig. S3. The expression profile of canonical and novel genes enriched in epithelial cluster identified by 10 × scRNA-seq. a, UMAP plot showing the epithelial cells in thyroid tissues could be clustered into parathyroid cells (PTC), parafollicular cells (C cell) and thyroid follicular cells (TFC). **b,** The percentage of the three epithelial subtype cells in thyroid tissues. **c,** The proportion of epithelial subtype cells in total epithelial cells in thyroid tissues at postnatal day 5, 10, 20 and 30 mice. **d,** Expression pattern of

canonical marker genes and DEGs that exclusively expressed in one subtype of PTC, C cell or TFC. Cells are colored by expression levels of these marker genes. Values are log transformed normalized UMIs for each marker gene in one single cell. **e,** Gene expression levels of these motility related genes (*Vim*, *Mcam* and *Map4k4*) were compared between TFC-1 and TFC-2 cell population. Data are shown as mean ± SD, and statistical significance was determined by two-sided Student's t test. **f,** UMAP of expression pattern of the Tnf-α-NF-κB pathway genes among TFC cells. Cells are colored by expression levels of the Tnf-α-NF-κB pathway genes. Values are log normalized UMI + 1. Source data are provided as a Source data file.

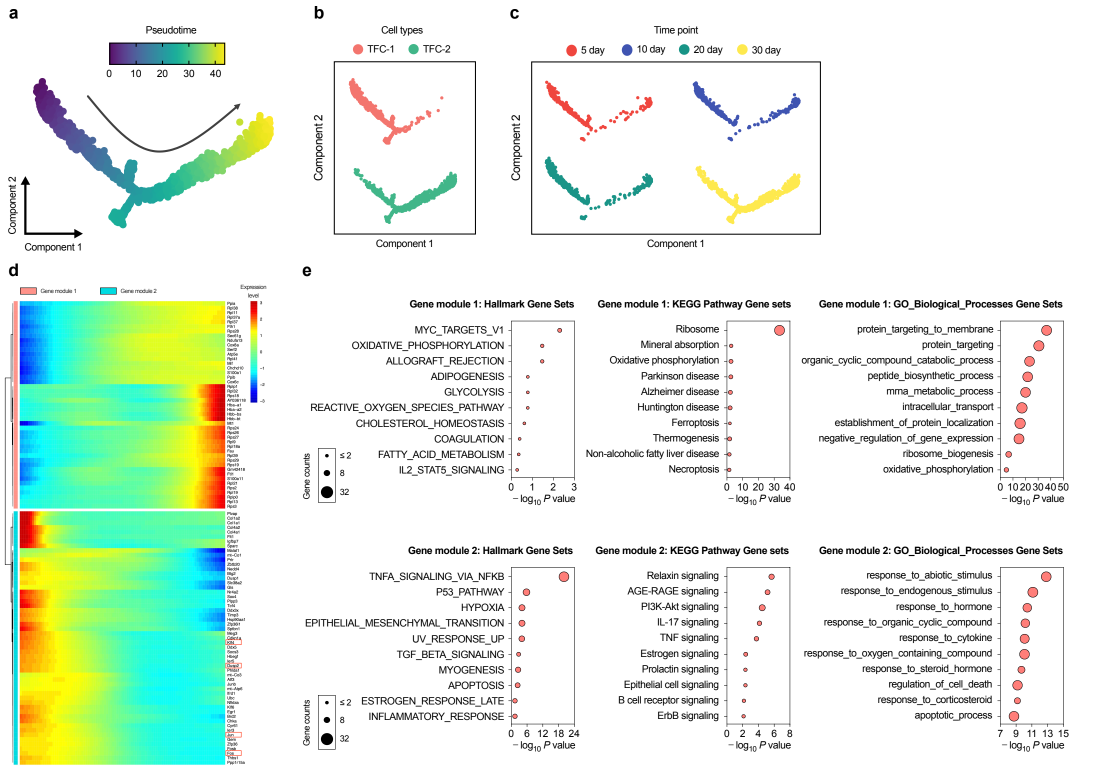

Fig. S4. Monocle trajectory analysis of the TFC cells.

a-c, Monocle2 prediction of the developmental trajectory of the TFC according to pseudo-time (**a**), cluster information (**b**) and developmental time (**c**). **d,** the DEGs (rows) along the pseudo-time (columns) were clustered hierarchically into two different modules. **e,** Hallmark gene sets, KEGG and GO analysis of the two gene modules. *P* value was determined by Benjamini-Hochberg-adjusted one-sided hypergeometric test. Source data are provided as a Source data file.

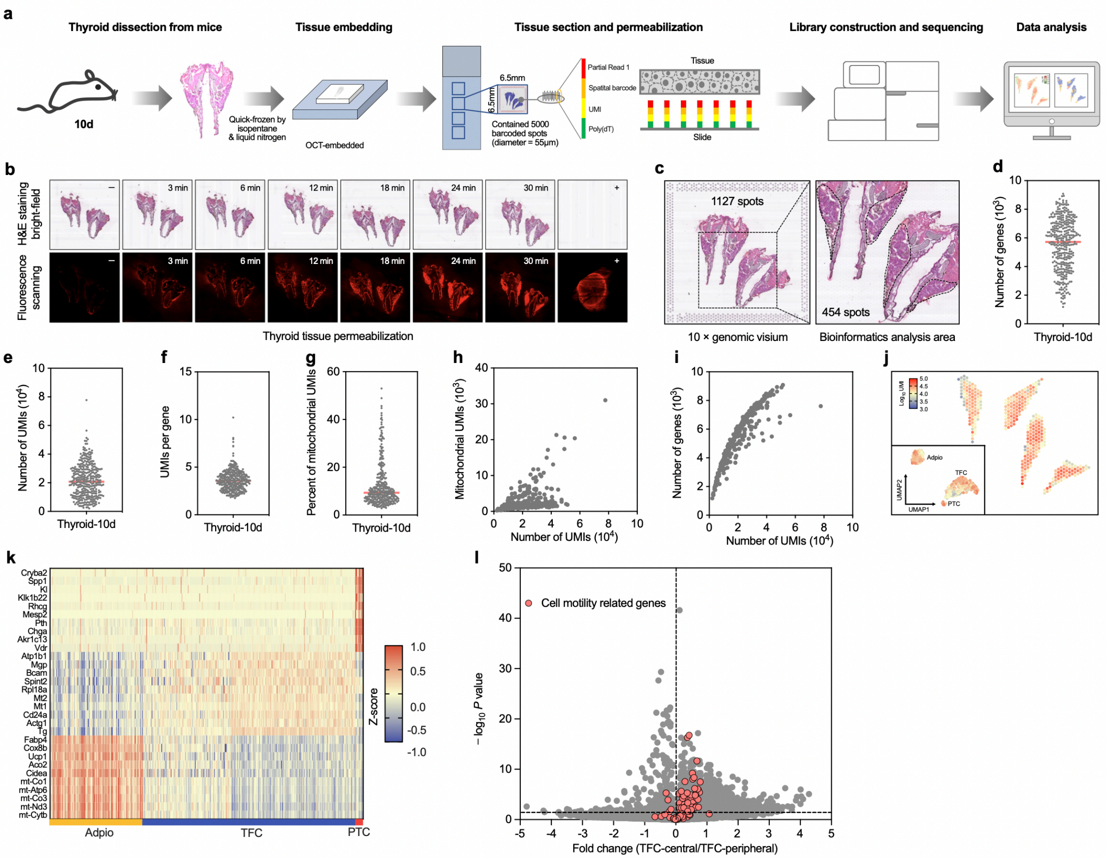

Fig. S5. ST of postnatal day 10 mice thyroid tissues. a, Schematic of the experimental design of the 10 × spatial transcriptome sequencing (ST-seq) in the thyroid tissues at postnatal day 10 mice. Cryosections of 2 thyroid glands at postnatal day 10 mice were mounted onto the capture area, which has ~5,000 barcoded spots. After tissue permeabilization and reverse transcription, cDNA library was constructed and subjected to sequence on Illumina Hiseq X Ten. **b,** Optimized permeabilization time (24 minutes) was obtained before generating Visium Spatial Gene Expression libraries. **c,** After quality control, the spatial transcriptome data from 1127 spots were retained. Out of the 1127 spots, 454 spots located in thyroid tissues of 2 postnatal day10 mice by H&E staining were subjected to further analysis. **d-g,** Sequencing and quality control metrics for ST-seq data. Distributions plots showed the spots with higher quality transcriptome data after removing the spots with low-quality transcriptome data. Number of unique genes identified from per spot from thyroid tissues at postnatal day 10 mice **(d)**, Number of UMIs identified from per spot in thyroid tissues at postnatal day 10 mice **(e)**, UMIs per gene **(f)** and percentage of UMIs that maps to a mitochondrial protein-encoding gene in per spot **(g)**. **h-i,** Dot plot showing the

relationship of the number of UMIs with mitochondrial UMIs **(h)** or with the number of genes **(i)** in the ST-seq data of per spot. **j,** Normalized UMI exhibit on the ST spots and UMAP embedding. **k,** Heatmap displaying top marker genes (y axis) in each subtype epithelial cell (x axis) based on differential expression genes testing. Color represents z-scored of the gene expression levels. **l,** Volcano plot showing the different expression genes between TFC-central and TFC-peripheral spots in thyroid tissues at postnatal day 10 mice. The red solid circles indicated the genes involved in the regulation of cell motility. *P* value was determined by two-sided Student's t test. Source data are provided as a Source data file.

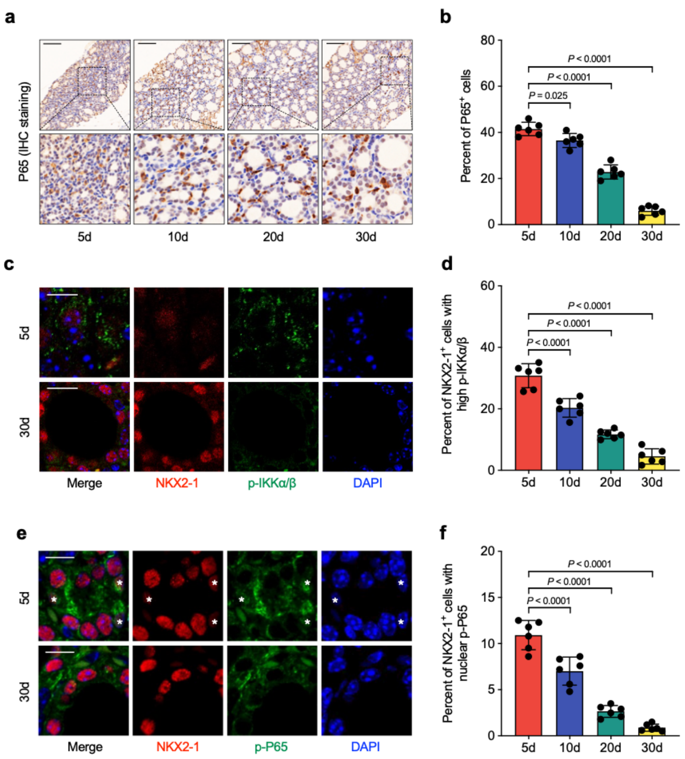

Fig. S6. The number of thyroid follicular cells with NF-κB activation gradually reduced in mice thyroid tissues from postnatal day 5 to day 30. a-b, Representative images **(a)** and statistical assessment **(b)** of P65 positive cells in thyroid tissues at postnatal day 5, 10, 20 and 30 mice by IHC analysis. **c,** Representative images showing the expression of phosphorylated IKKα/IKKβ in NKX-2-1 positive thyrocytes in the thyroid tissues of postnatal day 5 and day 30 mice by double immunofluorescence assay. **d,** Statistical assessment of the percentage of thyrocytes with high expression of P-IKKα/IKKβ in thyroid tissues at postnatal day 5, 10, 20 and 30 mice. NKX2-1 as a marker for thyrocyte. **e,** Representative images showing the expression of phosphorylated P65 (p-P65) in NKX2-1 positive thyrocytes in thyroid tissues at postnatal day 5 and 30 mice by double immunofluorescence assay. **f,** Statistical assessment of the percentage of nuclear p-P65 positive thyrocytes in thyroid tissues at postnatal day 5, 10, 20 and 30 mice. The number of p-P65 positive thyrocytes in thyroid tissues were gradually reduced with mice growth. Scale bar, 100 μm in **a**, and 25 μm in **c** and **e**. Data are shown as mean ± SD, n = 6 biologically independent samples, statistical significance was determined by One-way ANOVA, followed by Tukey's multiple comparison test (**b**, **d, f**). Source data are provided as a Source data file.

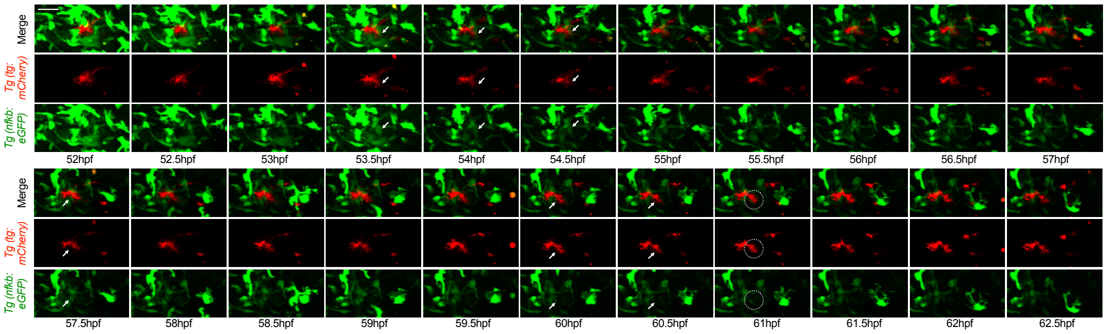

Fig. S7. NF-κB activation in thyrocytes during zebrafish folliculogenesis. Continuous observation of NF-κB activation during new follicles formation using zebrafish double transgenic *tg*:mCherry; *nfkb*:eGFP line, with time indicated on the bottom. White arrowheads (53.5 hpf, 54 hpf, 54.5 hpf) point to the front extended thyrocytes with NF-κB activation, and white arrowheads (60 hpf and 60.5 hpf) point to the rear migrated thyrocytes with NF-κB activation. From 61 hpf, when the epithelial cluster nearly separated from the TP (circled by white dotted lines) and a new follicle generated, the GFP fluorescent signals in these thyrocytes were faded down. Three independent experiments were carried. Scale bar: 50 μm.

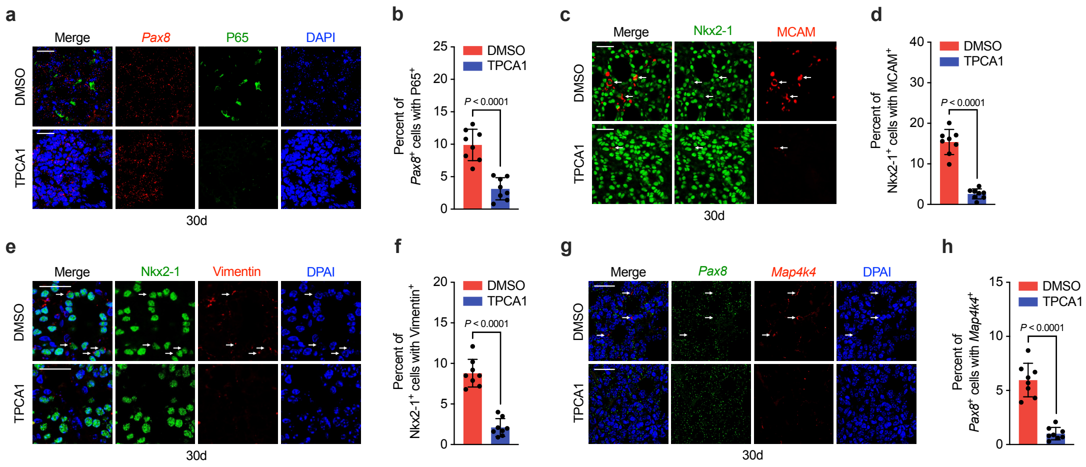

Fig. S8. TPCA1, an IKK2 inhibitor, suppressed NF-κB activation and motility of thyrocytes in mouse thyroid tissues. a-b, Representative image **(a)** showing the P65 and *Pax8* double positive thyrocytes and statistical assessment **(b)** of the percentage of P65/*Pax8* double positive cells in total *pax8* positive thyrocytes in thyroid tissues of mice with or without treatment by TPCA1. After treatment with TPCA1 for one month, the number of P65 and *Pax8* double positive thyrocytes were dramatically reduced in thyroid tissues at postnatal day 30 mice. **c-d,** Representative image **(c)** showing NKX2-1 and MCAM expression by double immunofluorescence assay and statistical assessment **(d)** of the percentage of NKX2-1/MCAM double positive cells in total NKX2-1 positive thyrocytes in thyroid tissues of mice with or without treatment by TPCA1. **e-f,** Representative image **(e)** showing NKX2-1 and Vimentin expression and statistical assessment **(f)** of the percentage of NKX2-1/Vimentin double positive cells in total NKX2-1 positive thyrocytes in thyroid tissues at postnatal day 30 mice with or without treatment by TPCA1. **g-h,** Representative image showing multicolor RNA scope staining of *Pax8*/*Map4k4* expression **(g)** and statistical assessment **(h)** of the percentage of *Pax8*/*Map4k4* double positive cells among total *Pax8* positive thyrocytes in thyroid tissues at postnatal day 30 mice with or without treatment by TPCA1. Scale bar, 50 μm. Data are shown as mean ± SD, n = 6 biologically independent samples, statistical significance was determined by One-way ANOVA, followed by Tukey's multiple comparison test (**b**, **d**, **f**, **h**). Source data are provided as a Source data file.

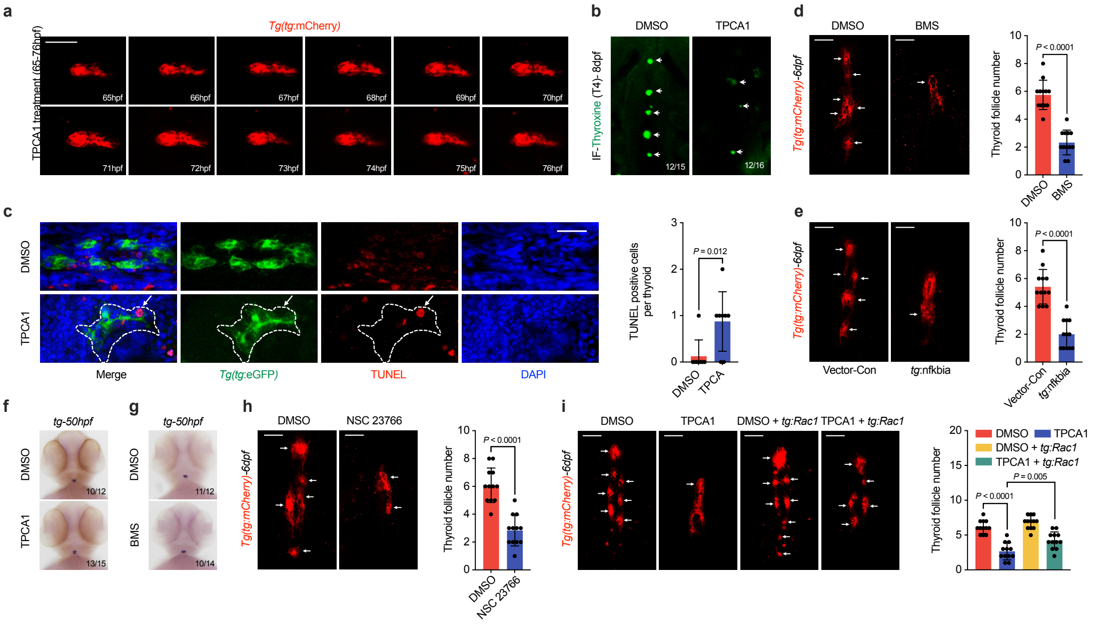

Fig. S9. NF-κB activation in thyrocytes is indispensable for zebrafish thyroid folliculogenesis. a, *In vivo* continuous observation of thyroid folliculogenesis during 65hpf to 76hpf zebrafish embryos treatment by TPCA1 in zebrafish transgenic *tg*:mCherry line. The thyrocytes extending out of the zebrafish thyroid primordium were suppressed and so the folliculogensis impeded in zebrafish embryos treatment by TPCA1. Three independent experiments carried. **b,** Representative images showing T4 positive follicles in 8dpf zebrafish embryos with or without TPCA1 treatment by IF staining. **c,** Representative image, and statistical assessment showing TUNEL positive cell per thyroid gland in 6dpf zebrafish embryos with or without TPCA1 treatment. **d,** Representative images and statistical assessment of thyroid follicle formed at 6 dpf embryos with or without treated by BMS-345541 using zebrafish transgenic *tg*:mCherry line. **e,** Representative images and statistical assessment showing thyroid specific inhibition of NF-κB activation by constitutively overexpression *nfkbia* under thyroglobulin promoter induced defective thyroid folliculogenesis of 6 dpf embryos using zebrafish transgenic *tg*:mCherry line. **f-g,** Comparison of *tg* expression in 50hpf WT and TPCA1 **(f)** or BMS **(g)** treated embryos by WISH. **h,** Representative image, and statistical assessment of follicles formed in zebrafish embryos treated with NSC23766 or DMSO. **i,** Representative image and statistical measurement of follicle numbers formed in 6dpf control and TPCA1 treated zebrafish embryos with or without

overexpression rac1 expression under *thyroglobulin* promoter. Scale bar, 50 µm. Data are shown as mean ± SD, n = 12 biologically independent samples, statistical significance was determined by Two-sided Student's t test (**d**, **c**, **e**, **h)**, or one-way ANOVA with post Tukey's multiple comparisons test (**i**). Source data are provided as a Source data file.

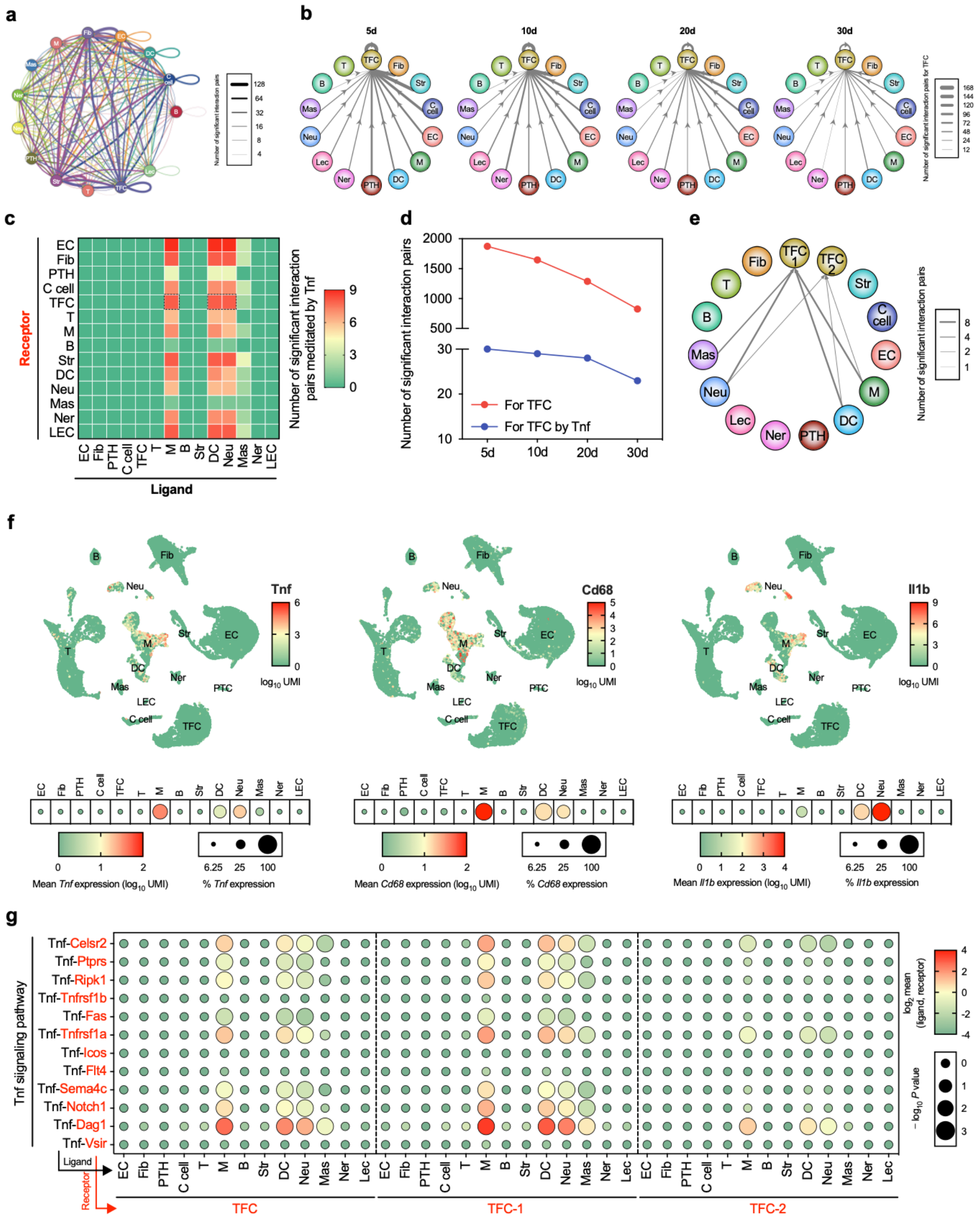

Fig. S10. The interaction of myeloid cells with thyrocytes identified by cellphoneDB mediated by Tnfα and its receptor pairs. a, Overview of the interaction pairs identified between 14 cell types identified by scRNA-seq. **b,** Ligand-receptor analysis to assess the number of interaction pairs against TFC cluster split by each time point. **c,** Numbers of significant interaction pairs between cell types mediated by Tnf. Colors indicate the numbers of significant interaction pairs. The x axis shows the cell type expressing the ligand Tnf and the y axis shows the cell type expressing the receptor.

Macrophage, dendric cells and neutrophils are the main types expressing the ligand Tnf. **d,** The transition trends of the number of significant total interaction pairs and Tnf mediated interaction pairs against TFC. **e,** The number of Tnf mediated interaction pairs toward TFC-1 and TFC-2 respectively. **f,** UMAP embedding of *Tnf*, *Cd68* and *Il1b.* Dot color intensity represents the log normalized UMI of expression values, and dot size represents percent of cells with at least one UMI detected per gene. **g,** Ligand-receptor analysis to assess TFC, TFC-1 and TFC-2 interactions with other cell types (right axis) mediated by Tnf. Specific ligand-receptor pairs are listed along y axis. Size of the dot indicates $-\log_{10} P$ value. Color of dots indicate interaction scores, which were calculated as the mean of the average log-normalized expression of ligand gene in one cell type and the average log-normalized expression of receptor gene in a second cell type. The thickness of the line represents the numbers of significant interaction pairs (**a**, **b**, **e**). Source data are provided as a Source data file.

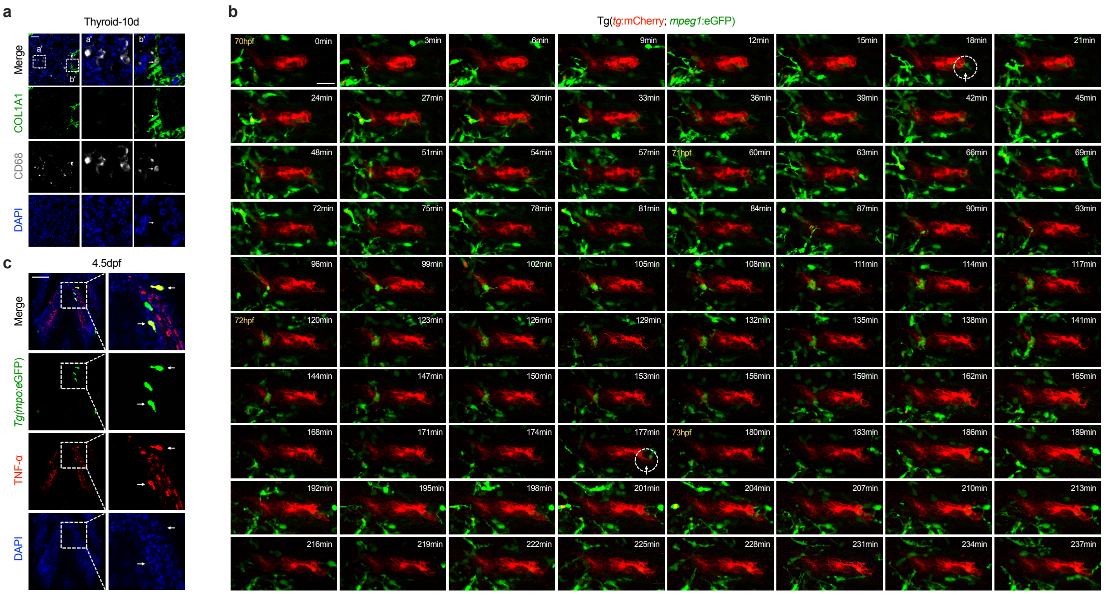

Fig. S11. Interactions of thyrocyte with myeloid cells promoted the migration of thyroid epithelial cells.

a, Myeloid cells are infiltrated into thyroid tissues at postnatal 10 mice. The panels of right two columns (a' and b') are enlarged pictures of areas framed on the left panel, which indicated CD68 positive macrophages infiltrated in the thyroid tissues at postnatal 10 mice. Arrows in (b') pointed to the CD68 positive macrophages located outside of the vascular stained by Col1a1. **b,** Long time continuous observation of the interaction with macrophages [Tg(*mpeg1*:eGFP)] promoted the collective migration of epithelial cluster [Tg(*tg*:mCherry)]. During this time window, obvious front epithelial cells migrations were detected, with one (18 minutes to 63 minutes) and another one (177 minutes to 216 minutes), and the start point were marked with white arrowhead and circled. Both epithelial migration events were accompanied with frequent interactions with surrounding macrophages. **c,** Tnf-α expression in neutrophils by immunofluorescence assays. Scale bar, 50 μm, and three independent experiments were carried for **a**, **b** and **c**.

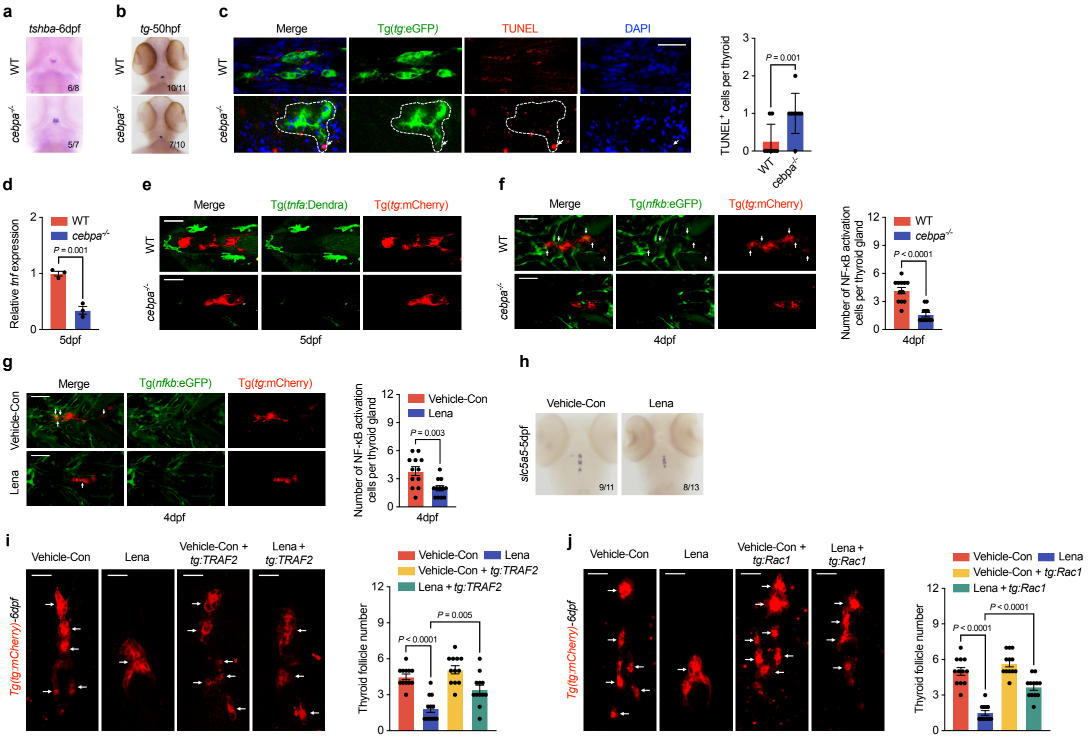

Fig. S12. Myeloid lineage and Tnf-α related signaling in promoting thyroid epithelial motility.

a, Representative image showing *tshba* expression in 6dpf WT or *cebpa* mutated zebrafish embryos by WISH. **b,** Comparison of *tg* expression in 50hpf WT and *cebpa* mutated zebrafish embryos by WISH. **c,** Representative image, and statistical assessment showing TUNEL positive cell per thyroid gland in 6dpf WT or *cebpa* mutated zebrafish embryos (n = 8). **d,** qPCR examination of the level of *tnf* transcripts in WT and *cebpa* mutants (n = 3). **e,** Tnf-α transgenic line was used to show *tnf* expression cells surrounding thyrocytes in WT and *cebpa* mutants. Three independent experiments were carried. **f,** Representative and statistical assessment of NF-κB activated thyrocytes in WT and *cebpa* mutants. Arrows pointed to the NF-κB activated thyrocytes observed (n = 12). **g,** Representative and statistical assessment of NF-κB activated thyrocytes in WT and Lenalidomide treated zebrafish embryos. Arrows pointed to the NF-κB activated thyrocytes observed (n = 12). **h,** Comparison of *slc5a5* expression in 6dpf control and lenalidomide treated zebrafish embryos by WISH. **i,** Representative images and statistical analysis of follicle numbers in control and lenalidomide treated embryos with or without TRAF2 overexpression (n = 12). **j,**

Representative images, and statistical analysis of follicle numbers in control and lenalidomide treated embryos with or without Rac1 overexpression in thyroid cells (n = 12). Scale bar, 50 μm. Data are shown as mean ± SD, and statistical significance was determined by Two-sided Student's t test (**c**, **d**, **f**, **g)**, or one-way ANOVA with post Tukey's multiple comparisons test (**i**, **j**). Source data are provided as a Source data file.

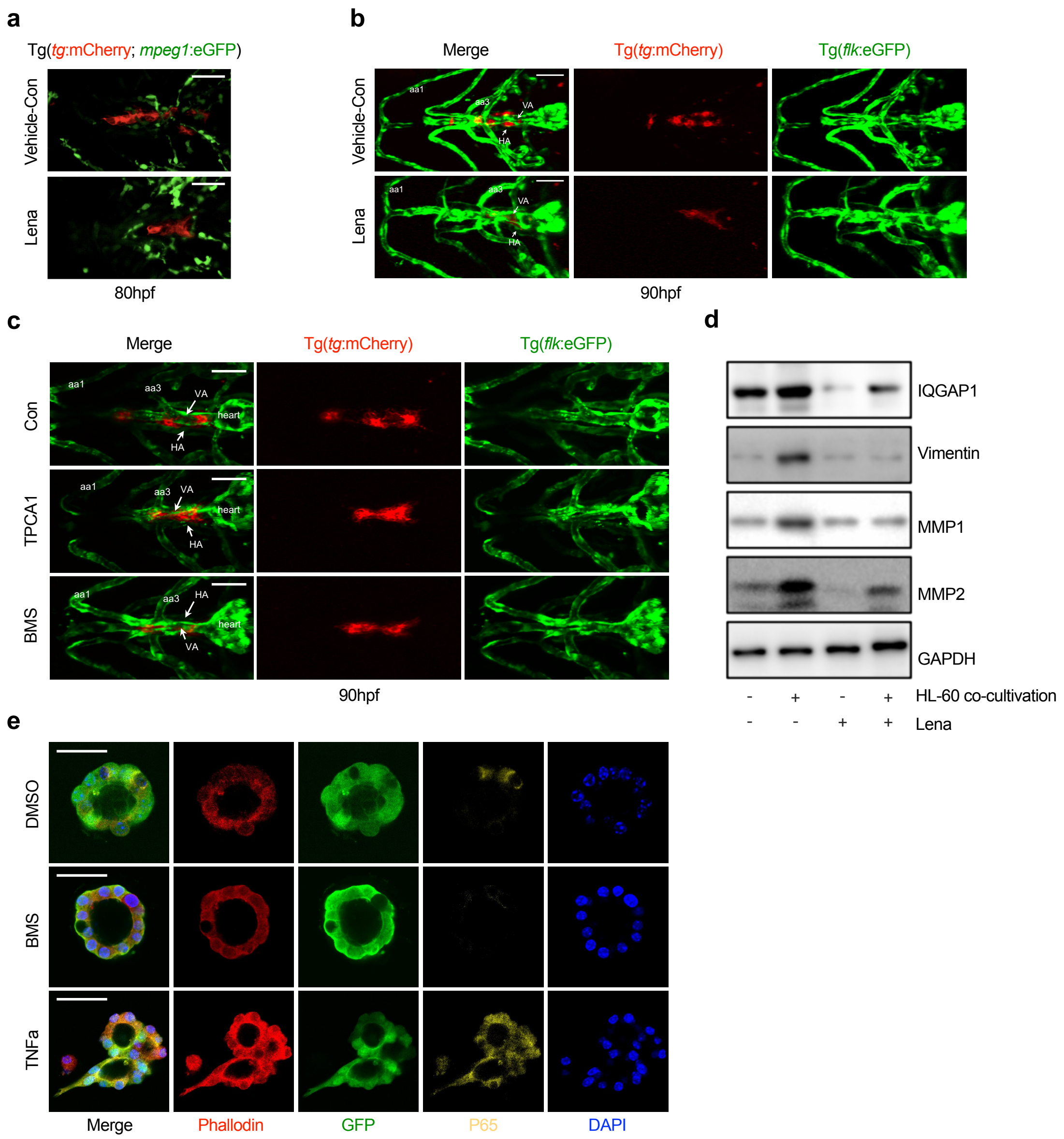

Fig. S13. Influence of Tnf-α and NF-κB signaling on zebrafish vascular development and mouse folliculogenesis *in vitro*. a, The influence of *mpeg1* positive macrophages surrounding TP with lenalidomide addition. **b-c,** Double transgenic line shows the vascular Tg(*flk*:eGFP) growth surrounding TP with lenalidomide, TPCA1 or BMS addition. **d,** Western blot assessment of migration related protein expression in Nthy-Ori3 normal thyroid epithelial cell line coculture with HL-60 or combined with lenalidomide treatment. **e,** Three-dimensional Matrigel for assessing folliculogenesis with mouse primary thyroid cells. The second row show little effect of BMS addition on follicle formation. The third panel show increased epithelial motility and muti-lumen formation induced with Tnf-α. Phalloidin for staining cytoskeleton and antibody against

phosphorylated P65 were used. Scale bar, 50 μm in **a**-**c**, and 25 μm in **e**. aa1, aa3, aortic arch arteries 1, 3; (H), HA, hypobranchial artery; VA, ventral aorta. Three independent experiments were carried for **a**-**e**.
