# Supplementary material for: Myeloid cells interact with a subset of thyrocytes to promote their migration and follicle formation through NF-κB
Source: Nat Commun. 2023 Dec 6;14:8082. doi: 10.1038/s41467-023-43895-8 (PMC10700497; doi:10.1038/s41467-023-43895-8)
Supplement: Supplementary file 3 — Reporting Summary [file 41467_2023_43895_MOESM3_ESM.pdf]

nature portfolio

Corresponding author(s): Huaidong Song
Last updated by author(s): Nov 22, 2023

Reporting Summary

Nature Portfolio wishes to improve the reproducibility of the work that we publish. This form provides structure for consistency and transparency in reporting. For further information on Nature Portfolio policies, see our Editorial Policies and the Editorial Policy Checklist.

Statistics

For all statistical analyses, confirm that the following items are present in the figure legend, table legend, main text, or Methods section.

n/a	Confirmed	
☐	☒	The exact sample size (*n*) for each experimental group/condition, given as a discrete number and unit of measurement
☐	☒	A statement on whether measurements were taken from distinct samples or whether the same sample was measured repeatedly
☐	☒	The statistical test(s) used AND whether they are one- or two-sided<br>*Only common tests should be described solely by name; describe more complex techniques in the Methods section.*
☐	☒	A description of all covariates tested
☐	☒	A description of any assumptions or corrections, such as tests of normality and adjustment for multiple comparisons
☐	☒	A full description of the statistical parameters including central tendency (e.g. means) or other basic estimates (e.g. regression coefficient) AND variation (e.g. standard deviation) or associated estimates of uncertainty (e.g. confidence intervals)
☐	☒	For null hypothesis testing, the test statistic (e.g. *F*, *t*, *r*) with confidence intervals, effect sizes, degrees of freedom and *P* value noted<br>*Give P values as exact values whenever suitable.*
☒	☐	For Bayesian analysis, information on the choice of priors and Markov chain Monte Carlo settings
☒	☐	For hierarchical and complex designs, identification of the appropriate level for tests and full reporting of outcomes
☒	☐	Estimates of effect sizes (e.g. Cohen's *d*, Pearson's *r*), indicating how they were calculated

Our web collection on statistics for biologists contains articles on many of the points above.

Software and code

Policy information about availability of computer code

Data collection	Image data was collected using NIS Elements (Nicon, V4.50.00)
Data analysis	For single cell RNA sequencing, data was processed using CellRanger (v3.0.1) recommended pipeline. Further analysis was conducted in R (V3.6.1) using Seurat (V3.1.1). Monocle2 (v2.8.0) in R was used to determine the pseudotimes of TFCs development. Cell-cell interaction was performed by CellphoneDB (V2.0). In non–scRNA-seq studies, data were presented as the mean ± standard deviation (SD). For GO analysis of TFC-central and TFC-peripheral cell types by using the Metascape tool (http://metascape.org). The statistical significance was tested by hypergeometric test and adjusted by Benjamini-Hochberg P value correction algorithm. For the ST data, genes with significantly higher expression in each spatial region relative to the others were identified (P < 0.001, two-tailed Student's t-test). With the specific gene sets extracted from the scRNA-seq and ST datasets, the overlap between each pair of cell type-specific and tissue region-specific gene sets was analyzed using MIA. The results were displayed using a hypergeometric test Group comparisons of normally distributed data were performed using an unpaired Student's t-test. One-way ANOVA, followed by Tukey's test was used for multiple groups comparison. Fisher's exact test was used for testing the independence of categorical data. Statistical analysis was performed in GraphPad Prism 7.04 (GraphPad Inc.).

For manuscripts utilizing custom algorithms or software that are central to the research but not yet described in published literature, software must be made available to editors and reviewers. We strongly encourage code deposition in a community repository (e.g. GitHub). See the Nature Portfolio guidelines for submitting code & software for further information.

Data

Policy information about availability of data

All manuscripts must include a data availability statement. This statement should provide the following information, where applicable:

- Accession codes, unique identifiers, or web links for publicly available datasets
- A description of any restrictions on data availability
- For clinical datasets or third party data, please ensure that the statement adheres to our policy

All data associated with this study are presented in the paper and the Supplementary Materials. This study did not generate any unique code. All software and algorithms used in this study are freely or commercially available and are listed in the Methods section. The sc-RNA seq data and the spatial seq data generated in this study are available in GEO database with accession code GSE231954 (https://www.ncbi.nlm.nih.gov/geo/query/acc.cgi?acc=GSE231954). The resulting fastq files were aligned to the mouse reference genome (mm10) (https://www.ncbi.nlm.nih.gov/datasets/genome/GCF_000001635.20/). Source data are provided with this paper.

Research involving human participants, their data, or biological material

Policy information about studies with human participants or human data. See also policy information about sex, gender (identity/presentation), and sexual orientation and race, ethnicity and racism.

Reporting on sex and gender	N/A
Reporting on race, ethnicity, or other socially relevant groupings	N/A
Population characteristics	N/A
Recruitment	N/A
Ethics oversight	N/A

Note that full information on the approval of the study protocol must also be provided in the manuscript.

Field-specific reporting

Please select the one below that is the best fit for your research. If you are not sure, read the appropriate sections before making your selection.

☒ Life sciences ☐ Behavioural & social sciences ☐ Ecological, evolutionary & environmental sciences

For a reference copy of the document with all sections, see nature.com/documents/nr-reporting-summary-flat.pdf

Life sciences study design

All studies must disclose on these points even when the disclosure is negative.

Sample size	No statistical method was used to predetermine sample size. Sample sizes were chosen based on sample availability and fundation.
Data exclusions	No data were excluded for analysis.
Replication	For each representative image/data, experiments were performed at least three times with similar results unless otherwise noted in the manuscript.
Randomization	All samples were randomly allocated into groups.
Blinding	Blinding was not possible as groups were divided by different treatments.

Reporting for specific materials, systems and methods

We require information from authors about some types of materials, experimental systems and methods used in many studies. Here, indicate whether each material, system or method listed is relevant to your study. If you are not sure if a list item applies to your research, read the appropriate section before selecting a response.

Materials & experimental systems

n/a	Involved in the study
☐	☒ Antibodies
☐	☒ Eukaryotic cell lines
☒	☐ Palaeontology and archaeology
☐	☒ Animals and other organisms
☒	☐ Clinical data
☒	☐ Dual use research of concern
☒	☐ Plants

Methods

n/a	Involved in the study
☒	☐ ChIP-seq
☒	☐ Flow cytometry
☒	☐ MRI-based neuroimaging

Antibodies

Antibodies used	NKX2-1(Servicebio, GB14157); vimentin (Servicebio, GB11192); MCAM (Invitrogen, 14-1469-82); P65 (CST, 8242); Phospho-IKKα/β (CST, 2697); Phospho-NF-κB p65 (Ser536) (CST, 3033); E-cadherin (Servicebio, GB12083); TPO (Servicebio, GB14160); Thyroxine (Abcam, ab30833); CD68 (Servicebio, GB113109); IL-1 beta (Servicebio, GB11113);TNF-α (Servicebio, GB11188); IQGAP1(Invitrogen, 33-8900); Vimentin (Servicebio, GB11192); MMP1 (Thermo, PA5-115581); MMP2 (Servicebio, GB11130);GAPDH (Sigma, WH0002597M1)
Validation	Anti-Anti-TTF1(Homeobox protein Nkx-2.1) Mouse mAb, Servicebio, Cat# GB14157. Supplier validation: "Species reactivity, Reacts with: Human, applications Suitable for: IHC, IF".<br>Anti -Vimentin Rabbit pAb, Servicebio, Cat# GB11192, Supplier validation: "Species reactivity, Reacts with: Human, Mouse, Rat, applications Suitable for: IHC, IF,WB".<br>Anti -CD146 Monoclonal Antibody (P1H12), eBioscience, Invitrogen, Cat# 14-1469-82, Supplier validation: "Species reactivity, Reacts with: Dog, Human, Mouse, Rabbit, applications Suitable for: ICC/IF, Flow Cyto, WB, IHC-P".<br>Anti -NF-κB p65 (D14E12) XP® Rabbit mAb, CST, Cat#8242, Supplier validation: "Species reactivity, Reacts with: Human, Mouse, Rat, applications Suitable for: ICC/IF, Flow Cyt (Intra), WB, IHC-P".<br>Anti -Phospho-IKKα/β (Ser176/180) (16A6) Rabbit mAb, CST, Cat#2697, Supplier validation: "Species reactivity, Reacts with: Human, Mouse, Rat, applications Suitable for: WB, IHC-P".<br>Anti -Phospho-NF-κB p65 (Ser536) (93H1) Rabbit mAb, CST, Cat#3033, Supplier validation: "Species reactivity, Reacts with: Human, Mouse, Rat, applications Suitable for: WB, IHC-P,IF".<br>Anti -E Cadherin Mouse mAb, Servicebio, Cat#GB12083, Supplier validation: "Species reactivity, Reacts with: Human, Mouse, Rat, applications Suitable for: IHC/IF".<br>Anti -Thyroid Peroxidase/TPO Mouse mAb, Servicebio, Cat#GB14160, Supplier validation: "Species reactivity, Reacts with: Human, applications Suitable for: IHC/IF".<br>Anti-Thyroxine antibody,Abcam, Cat#ab30833,Supplier validation: "Species reactivity, Reacts with: Human, , applications Suitable for: ELISA,RIA".<br>Anti -CD68 Rabbit pAb, Servicebio, Cat#GB113109, Supplier validation: "Species reactivity, Reacts with: Mouse, Rat, applications Suitable for: IHC/IF".<br>Anti -TNF-alpha Rabbit pAb, Servicebio, Cat#GB11188, Supplier validation: "Species reactivity, Reacts with: Mouse, Rat, applications Suitable for: IHC/IF".<br>Anti -IQGAP1 Monoclonal Antibody (AF1), Invitrogen,Cat#33-8900, Supplier validation: "Species reactivity, Reacts with: Human, Mouse, Non-human primate, applications Suitable for: WB, IP, IF and ELISA".<br>Anti -Vimentin Rabbit pAb, Servicebio, Cat#GB11192, Supplier validation: "Species reactivity, Reacts with: Human, Mouse, Rat, applications Suitable for: WB, IHC, IF ".<br>Anti -MMP1 Polyclonal Antibody,Thermo, Cat#PA5-115581, Supplier validation: "Species reactivity, Reacts with: Human, applications Suitable for: WB, ICC/IF ".<br>Anti -MMP2 Rabbit pAb Rabbit pAb, Servicebio, Cat#GB11130, Supplier validation: "Species reactivity, Reacts with: Human, Mouse, Rat, applications Suitable for: WB, IHC, IF ".<br>Anti -Monoclonal Anti-GAPDH antibody produced in mouse, Sigma, Cat#WH0002597M1, Supplier validation: "Species reactivity, Reacts with: Human, applications Suitable for: WB ".

Eukaryotic cell lines

Policy information about cell lines and Sex and Gender in Research

Cell line source(s)	Nthy-Ori3 (Sigma, 90011609) and HL60 cells (Sigma, 98070106) were used.
Authentication	The cell-lines were not authenticated.
Mycoplasma contamination	All cell lines were tested negative for mycoplasma.
Commonly misidentified lines (See ICLAC register)	None of the cell lines is listed in the ICLAC Register of misidentified lines.

Animals and other research organisms

Policy information about studies involving animals; ARRIVE guidelines recommended for reporting animal research, and Sex and Gender in Research

Laboratory animals	Wild-type (WT) mice were obtained from Cyagen Biosciences, China. All mice were housed in a pathogen-free environment with the temperature maintained at 23 ± 2 °C and relative humidity at 50 to 65% under 12 hours light/dark cycle. Mice were fed with normal chow (cat:19123123, Beijing KeaoXieli Feed Co., Beijing, China) with free access to food and water. Mice were euthanized with a combination of CO_2 and cervical dislocation to guarantee the death of the animals. Zebrafish were housed in the zebrafish center of Shanghai Ninth People's Hospital. 5-20 adult mixed-sex fish per liter were grown in water with temperature, pH, and conductivity monitored daily and maintained at 26–29° C, 7–8, and 200–3000 micro-Siemens, respectively. Live brine shrimp in addition to standard diet were used to feed adult fish. Zebrafish maintenance and staging were performed using standard protocols.Transgenic zebrafish lines mpeg1:eGFP, mpo:eGFP, flk1: eGFP and tg:eGFP were obtained from China zebrafish resource center. Tg:mCherry was created by our lab (reference seen in the manuscript). The cebpaΔCA zebrafish lines was a gift from Jun Zhu's lab (reference seen in the manuscript). The plasmid used for creating Tg(nfkb: eGFP) was a gift from John Rawls (Addgene plasmid # 44922). For Tg(tnfa: dendra) transgenic lines, the TNFa promoter (Gene ID: 405785) was amplified from zebrafish genomic DNA and then cloned into Tol2 vector backbone (Addgene, plasmid # 51462) upstream of dendra2 (reference seen in the manuscript). The plasmids were linearized and co-injected with transposon mRNA at the zebrafish one-cell stage. Stable transgenic lines were finally obtained by offspring fluorescence screening.
Wild animals	No wild animals were used.
Reporting on sex	Sex was not considered in this study design since our preliminary experiments showed no differences of thyroid development based on sex.
Field-collected samples	The study did not involve samples collected from the field.
Ethics oversight	The study was approved by the ethics committee of Shanghai Ninth People's Hospital.

Note that full information on the approval of the study protocol must also be provided in the manuscript.
